# Optimization of Droplet Granulation Process for HNS-IV Explosives Utilizing Pulsed Air-Jet Shear Technology

**DOI:** 10.3390/molecules31061058

**Published:** 2026-03-23

**Authors:** Yuruo Zhang, Jinbo Liu, Peng Zhu, Jingyu Wang

**Affiliations:** 1 School of Environment and Safety Engineering, North University of China, Taiyuan 030051, China; 2 Shaanxi Applied Physics and Chemistry Research Institute, Xi’an 710061, China; 3 School of Chemistry and Chemical Engineering, Nanjing University of Science and Technology, Nanjing 210094, China

**Keywords:** droplet microfluidics, pulsed air-jet, composite microspheres, active control, frequency-controlled preparation

## Abstract

To achieve precise control over droplet size and generation frequency in the granulation process of HNS-IV, this study introduces a novel droplet granulation strategy that utilizes pulsed air-jet shearing technology. This approach enables independent and precise regulation of droplet injection frequency (fg) and volume (V) through systematic adjustments of air pressure (P), frequency (fp), duty cycle (η), and liquid flow rate (Q). By controlling the suspension flow rate (Q), we successfully achieved primary particle size control, obtaining median particle sizes (D50) of 375.84 μm, 444.45 μm, and 504.22 μm in ascending order. Furthermore, we systematically investigated the influence of calcium alginate (CA) concentration on both the sphericity of the resultant particles and the thermal decomposition characteristics of HNS microspheres. Our findings demonstrate that while increased CA content enhances particle sphericity, it simultaneously affects the thermal decomposition behavior of the microspheres. The proposed pulsed air-jet shearing method offers significant advantages by significantly reducing the accumulation of volatile organic solvents typical of liquid–liquid biphasic systems. Furthermore, the residual non-toxic aqueous solutions can be easily managed, establishing a greener, safer, and highly controllable approach for HNS-IV granulation. This methodology presents a valuable reference for achieving precise and controllable granulation of various energetic materials.

## 1. Introduction

Hexanitrostilbene (2,2′,4,4′,6,6′-hexanitrodiphenylethylene, HNS) is a high-energy density compound known for its excellent explosive performance [1]. As the primary explosive used in heat-resistant transfer explosives, the structural morphology, particle size, and mixing uniformity of HNS are critical factors influencing its energy release characteristics [2,3,4]. However, the high surface energy and low sphericity of nano-scale HNS-IV powder make it susceptible to aggregation, necessitating granulation to enhance its dispersibility. Currently, conventional granulation methods, such as water suspension [5,6] and spray drying [7,8], have been well-established for macroscopic granulation processes. However, these methods often result in poor batch-to-batch consistency, uneven mixing, and low sphericity of the explosives.

In recent years, droplet microfluidics technology has rapidly advanced in the field of energetic material granulation [9,10]. The fundamental principle involves the formation of micro-droplets through the shear action of immiscible phases within microchannels, utilizing droplets as templates for the spherical granulation of energetic materials. Shi Yu et al. [11,12,13] successfully achieved the continuous and controllable preparation of spherical propellant using a T-shaped microchannel with a water/ethyl acetate system. Han [14] and Liu [15,16,17] employed droplet flow to attain spherical granulation of various energetic materials, including Octogen (HMX) and HNS. Zhou prepared microscale boron/chromium barium delay powder particles using a microarray chip [18]. To effectively encapsulate these energetic materials and form composite microspheres, alginate-based gelation has emerged as a promising route [19]. Traditional alginate-based microsphere production typically relies on simple dripping/extrusion methods, which suffer from large particle sizes (usually >1 mm) and low throughput. Electrospray or electrostatic droplet generation can produce finer particles with narrow polydispersity, but their application is often severely limited by the high viscosity and complex electrical conductivity of dense energetic particle suspensions. While conventional closed-channel microfluidics (such as flow-focusing or T-junctions) offer excellent size control, they are highly prone to channel clogging when processing high-solid-loading slurries like HNS-IV, and droplet generation is heavily influenced by fluid viscosity and channel dimensions.

As an important branch of droplet microfluidics technology, droplet jetting technology utilizes airflow to shear the dispersed phase and employs an open structure to directly collect and solidify the generated droplets outside the microchannel using a container [20]. This structure helps to mitigate particle blockages that may occur in closed microchannels [21], thereby enhancing system stability [22]. Compared to liquid–liquid biphasic flow, the droplet jetting process based on airflow shearing minimizes liquid waste accumulation, facilitating a greener granulation process. However, in the droplet jetting process under continuous airflow, controlling droplet volume depends on airflow pressure and liquid flow rate. While regulating droplet volume, these parameters indirectly affect the jetting frequency and speed of the droplets [23,24,25], making it challenging to independently and precisely control the droplet jetting frequency [26,27].

This paper proposes a method for the spherical granulation of HNS-IV based on pulse airflow shearing of droplets. An external disturbance source is introduced to generate pulse airflow, which shears the liquid phase to form micro-droplets. The jetting frequency of these micro-droplets can be controlled by the frequency of the pulse airflow, thereby achieving the spherical granulation of HNS-IV.

## 2. Results and Discussion

### 2.1. Droplet Breakup Mechanism Under Airflow Shearing

**Figure 1:**
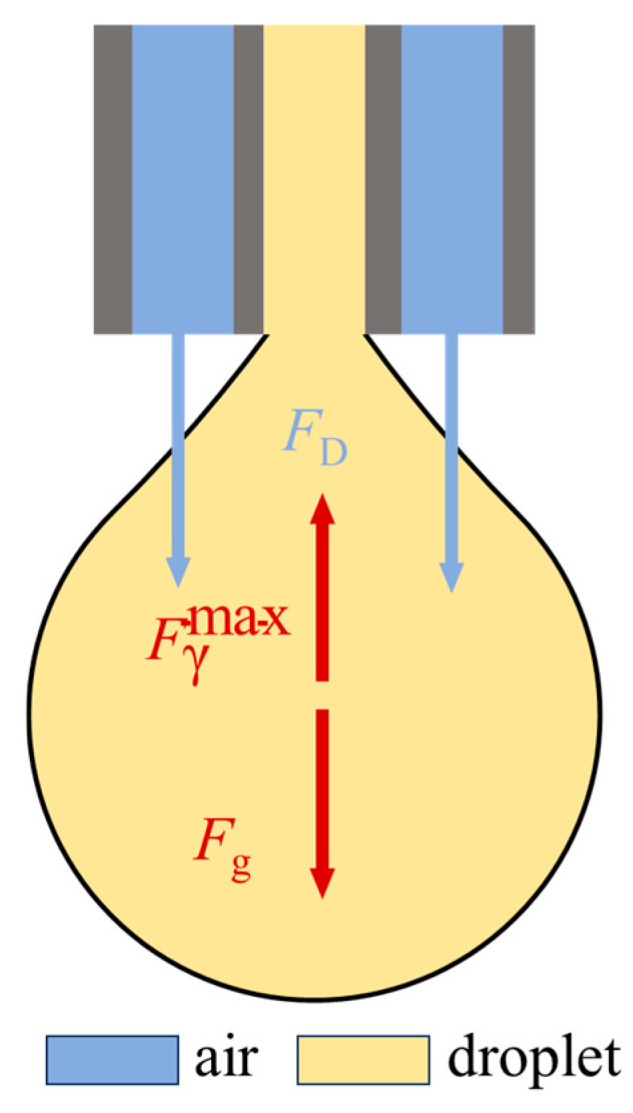
The force situation during droplet growth process. The red arrows indicate the direction of the forces, and the blue arrows represent the direction of the continuous airflow.

Similar to the concept of differentiation, continuous airflow can be regarded as the limiting state of pulsed airflow when its frequency approaches infinity. We first discuss the droplet breakup process under the influence of continuous airflow. The forces acting on the droplet during its growth are shown in Figure 1. According to Tate’s law, in the quasi-static limit, a droplet will detach from the nozzle when the gravitational force *F_g_* exceeds the maximum capillary force $F_{\gamma }^{\max}$ [28].

(1) $$F_{g}>f_{HB}F_{\gamma }^{max}$$

where *f_HB_* is a constant less than 1, referred to as the Harkins-Brown correction factor, which accounts for the liquid that remains attached to the nozzle after droplet detachment. Under the influence of continuous airflow, the droplet experiences a downward shear force *F_D_*. For droplet detachment to occur, the following condition must be satisfied:

(2) $$F_{g}\;+\;F_{D}\;>\;f_{\mathrm{HB}}F_{\gamma }^{\max}$$

Under a given fluid flow rate *Q*, the accumulated droplet volume *V*(*T*) increases linearly with time (*V*(*T*) = *Q T*). Thus, *F_g_* varies linearly with time and can be expressed as:

(3) $$F_{g}\;={\;\rho }_{l}\mathrm{QgT}$$

where *ρ*_l_ is the fluid density, *Q* is the fluid flow rate, *g* is the gravitational acceleration, and *T* is time. *F_D_* is given by the following expression [29]:

(4) $$F_{D}\;=\;0.5C_{D}Au^{2}\rho_{g}$$

where *C_D_* is the drag coefficient, *ρ_g_* is the gas density, and *A* represents the cross-sectional area of the droplet exposed to the airflow, which can be expressed as *A* = 1/4πd^2^. Therefore, as the droplet diameter increases, the droplet will detach from the nozzle when the three forces satisfy Equation (2).

Many studies have interpreted the entire process of droplet detachment as the result of *F_g_* and *F_d_* overcoming the maximum capillary force $F_{\gamma }^{\max}$ [30,31]. In this perspective, it is suggested that the droplet is “pulled apart” by *F_g_* and *F_D_*, with these forces being regarded as the dominant factors leading to droplet breakup. However, through our investigation of droplet ejection under pulsed airflow, we observed that the droplet does not immediately break when subjected to the instantaneous shear force (*F_D_*); instead, there is a slight delay. This suggests that under pulsed airflow conditions, the dominant force ultimately causing droplet breakup is not *F_g_* or *F_D_*. The behavior of fluid flow always follows the principle of minimum energy. The growth, stretching, and breakup of a droplet adhere to the law of energy conservation. When the droplet first begins to form from the nozzle, surface tension acts to retract the droplet back to the nozzle in order to minimize surface energy. Under the combined effects of pump thrust, *F_g_* and *F_d_*, the droplet volume increases, elongates, and gradually displaces downward. This process leads to the accumulation of energy released from surface tension (surface energy of the fluid). When this energy release accumulates to a critical threshold, the elongated fluid, instead of retracting to the nozzle, tends to contract independently into a single droplet to achieve the lowest system energy. Therefore, under the influence of pulsed airflow, we propose that it may be more accurate to define the direct force leading to droplet breakup as tension. The work performed by *F_g_*, *F_d_* and any other forces on the droplet ultimately transforms into the surface energy of the fluid.

To quantitatively characterize the dynamic regime of the droplet break-up process under pulsed airflow, dimensionless numbers including the Weber number (*We*), Reynolds number (*Re*), and Ohnesorge number (*Oh*) were evaluated. The Weber number (*We* = *ρ_g_⋅u*^2^⋅*d*/*σ*) describes the relative importance of aerodynamic shear forces compared to surface tension. In our system, the localized high-velocity pulsed gas jet yields a high *We* regime, indicating that aerodynamic drag provides the primary energy to stretch the droplet. The Reynolds number (*Re* = *ρ_g_⋅u⋅d*/*μ_g_*) further confirms the turbulent nature of the shearing airflow. Concurrently, the Ohnesorge number (*Oh* = *μ*_l_/$\sqrt{\rho_{l}\sigma d}$) relates the viscous forces of the sodium alginate (SA) suspension to inertial and surface tension forces. The relatively high viscosity of the suspension results in an elevated *Oh* number, which effectively dampens immediate aerodynamic shattering (secondary break-up) and ensures the formation of intact primary droplets. Therefore, while *F_g_* and *F_D_* initiate the stretching deformation, the final detachment timing and pinch-off are ultimately governed by the capillary action driven by the minimization of surface energy within the pulse duration.

### 2.2. Achieving Controlled Droplet Ejection in Single-Periodic Mode

The droplet ejection technology based on pulsed airflow enables precise and independent control over the frequency (*f_g_*) and volume (*V*) of droplet ejection by regulating the pressure (*P*) of the pulsed airflow, the driving frequency (*f_p_*), the duty cycle (*η*), and the liquid flow rate (*Q*). The droplet volume is generally determined by the following equation:

(5) $$V\;=\;\frac{Q}{f_{g}}$$

By adjusting *P*, *Q*, *f_p_*, and *η*, three distinct droplet generation modes can be observed: sub-periodic, single-periodic, and multi-periodic. During the ejection process, in the single-periodic mode, the droplet ejection frequency (*f_g_*) matches the driving frequency (*f_p_*) (i.e., *f_g_* = *f_p_*). Under the instantaneous strong shear force exerted by the pulsed airflow in each cycle, droplets detach stably and orderly. In the single-periodic mode, fg can be freely regulated by *f_p_*, enabling linear control of the droplet volume, as expressed by the following relationship:

(6) $$V\;=\;\frac{Q}{f_{p}}$$

**Figure 2:**
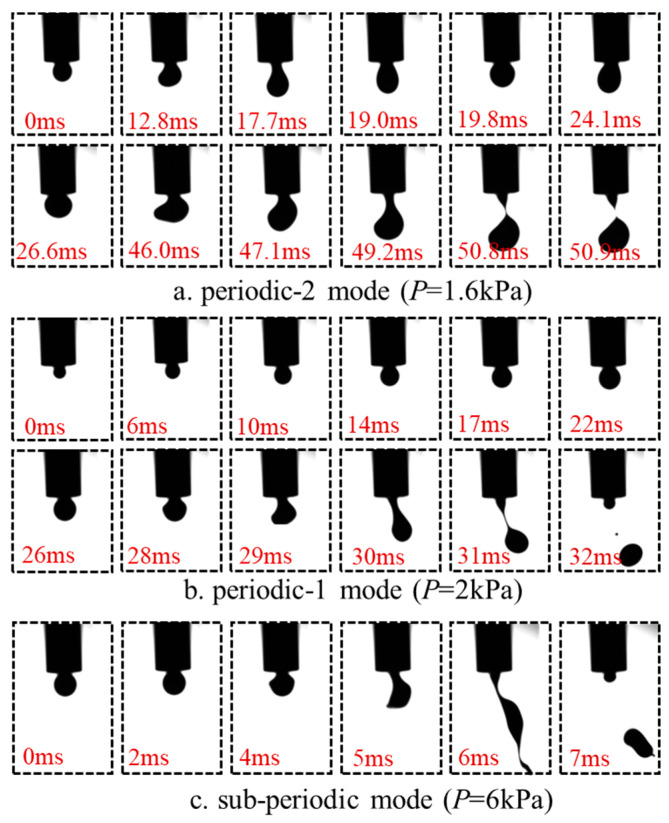
Three jetting modes exhibited by droplets under the action of pulsed airflow.

However, the discrete process of suspension-phase fluids containing multiple solid components exhibits unique characteristics, involving the coupling effects of multiphase fluids such as the aqueous phase and solid phase, resulting in complex flow behaviors. Achieving high-frequency ejection of suspension-phase droplets in the single-periodic mode is particularly challenging. To investigate the droplet ejection behavior under pulsed airflow, we maintained a duty cycle (*η*) of 50%, a liquid flow rate (*Q*) of 600 μL/min, and a driving frequency (*f_p_*) of 30 Hz. By adjusting the air pressure (*P*), we observed three distinct ejection modes of droplets under the shear action of the pulsed airflow, as illustrated in Figure 2.

At an air pressure of *P* = 1.6 kPa, the droplet undergoes a total of 50.9 ms from its formation to its detachment, during which the droplet generation frequency (*f_g_*) is 19 Hz, which is less than the driving frequency (*f_p_*). Under these conditions, the droplet requires at least one shear action from the pulsed airflow to achieve final detachment, corresponding to the aforementioned multi-periodic mode (periodic-n mode): *f_g_* < *f_p_*, as shown in Figure 2a. When the air pressure is adjusted to 2 kPa, the droplet generation frequency (*f_g_*) becomes 30 Hz, matching the driving frequency (*f_p_*). Under these parameters, droplets detach smoothly each time the airflow shear is applied. The droplet generation frequency aligns with the frequency of the pulsed airflow, representing the single-periodic mode (periodic-1 mode): *f_g_* = *f_p_*, as illustrated in Figure 2b. Experiments reveal that droplets can be stably ejected in the single-periodic mode within a certain range of air pressure. However, when the air pressure is excessively high (*P* = 7 kPa), the overly strong shear force fragments the droplets into two or even multiple smaller droplets, introducing additional instability to the ejection process and the final size distribution. This is one of the reasons for droplet ejection in the sub-periodic mode (sub-periodic mode, *f_g_* > *f_p_*), as depicted in Figure 2c.

To quantitatively highlight the advantages of this pulsed air-jet strategy, it is essential to compare it with conventional continuous air-blast microfluidic systems (as described in References [27,28,29,30]). In continuous airflow systems, the droplet detachment relies on a passive force balance where aerodynamic drag dynamically overcomes capillary forces. Consequently, the droplet generation frequency (*f_g_*) and volume (*V*) are strongly coupled; adjusting the airflow pressure (*P*) to change droplet size inevitably alters the frequency in a non-linear manner, often leading to a wider particle size distribution (Coefficient of Variation, CV, typically >5%). In stark contrast, the proposed pulsed air-jet introduces an active perturbation mechanism. In the single-periodic mode (*f_g_* = *f_p_*), the temporal detachment of the droplet is strictly locked to the driving frequency. This active decoupling allows the precise calculation of droplet volume purely based on the fluid flow rate and pulse frequency (*V* = *Q*/*f_p_*), achieving highly monodisperse energetic microspheres with excellent dimensional predictability that passive continuous systems struggle to attain.

### 2.3. The Influence of Different Sodium Alginate (SA-ω) Concentrations on Microsphere Morphology

**Figure 3:**
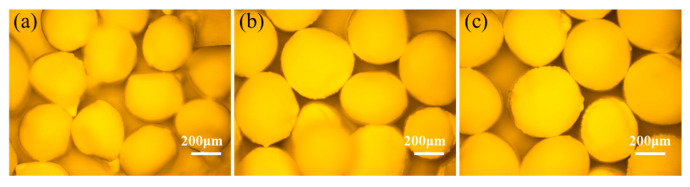
Optical micrograph of HNS microspheres (**a**) SA—0.6%, (**b**) SA—0.8%, (**c**) SA—1.0%.

In the process of jet granulation, the concentration of SA governs the solidification reaction rate of microdroplets, making it a critical factor influencing the sphericity quality of the particles. Under the single-periodic mode, jet granulation of HNS was conducted with experimental parameters set at *η* = 50%, *Q* = 600 μL/min, *f_p_* = 30 Hz, and an air pressure of 2.2 kPa. Optical microscopy was employed to observe droplets at varying SA concentrations, with the results depicted in Figure 3. The SA concentration (ω) is defined rigorously as the mass ratio of SA to the total mass of the solution. A higher SA concentration facilitates improved sphericity of the microdroplets. When the SA concentration is below 0.8%, the microdroplets tend to develop a “tailing” structure (quantitatively defined as an aspect ratio *L*/*W* > 1.2 with an asymmetrical tapered end), attributed to the slow gelation process at lower SA concentrations. The solidification reaction of the droplets initiates upon contact with the calcium chloride solution interface. A higher SA concentration accelerates the gelation process, helping the droplets maintain their original spherical morphology as they traverse the interface. Conversely, at lower concentrations, the slower gelation process results in semi-solidified microcapsules that form tailing structures under the influence of interfacial tension.

**Figure 4:**
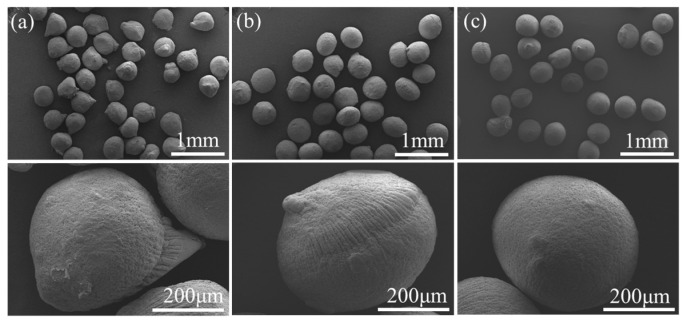
SEM image of HNS microspheres (**a**) SA—0.6%, (**b**) SA—0.8%, (**c**) SA—1.0%.

The solidified capsule microspheres were heated and dried to obtain HNS microparticles, with SEM images of the microparticles shown in Figure 4. The SA concentration significantly influences the surface smoothness of the particles. At lower SA concentrations, the reduced calcium alginate (CA) content within the microspheres leads to pronounced component aggregation during the drying process, resulting in a surface characterized by distinct trench-like structures. Furthermore, the SEM images from the three groups indicate that the SA concentration does not markedly affect the particle size of the dried HNS microparticles.

### 2.4. Analysis of the Internal Structure of Microspheres

**Figure 5:**
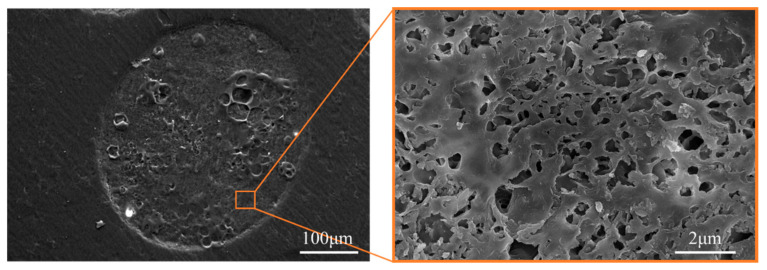
Microsphere slice image.

Ion milling technology utilizes an ion beam to etch the surface of a sample, gradually thinning it to enable high-precision characterization of its fine structure. This technique is applicable to a wide range of materials. To observe the internal structure of HNS microspheres, ion milling was utilized to obtain microsphere cross-sections, as illustrated in Figure 5. The results reveal that the composite microparticles exhibit a relatively dense internal structure. However, due to the cross-linking reaction between SA and calcium chloride, the interior of the microspheres presents a networked structure, resulting in the presence of pores within the final microspheres.

**Figure 6:**
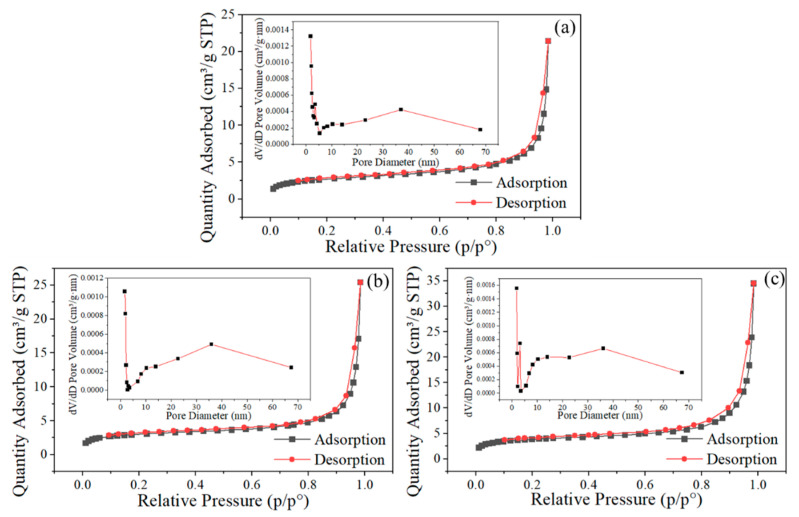
N_2_ adsorption desorption curves of microspheres at different SA concentrations (**a**) SA—0.6%, (**b**) SA—0.8%, (**c**) SA—1.0%. The black lines with square symbols represent the adsorption curves, and the red lines with circular symbols represent the desorption curves.

To further quantitatively characterize the surface area and internal porosity of the microspheres, BET analysis was performed, as shown in Figure 6. The results indicate that the N_2_ adsorption–desorption curves of the four samples are all typical Type IV isotherms, characterized by hysteresis during the adsorption–desorption process and the formation of a hysteresis loop following capillary condensation. The specific surface areas of the HNS microparticles prepared at SA concentrations of 0.6%, 0.8%, and 1.0% were 9.7750 m^2^/g, 11.2250 m^2^/g, and 13.9445 m^2^/g, respectively. The average pore diameters were 27.8770 nm, 27.8442 nm, and 26.7987 nm, respectively. Despite minimal differences in average pore diameter, the specific surface area of the microspheres significantly increases with higher SA concentrations. At elevated SA concentrations, the cross-linking reaction results in a more densely networked gel structure, which helps to reduce the aggregation of nano-components within the microspheres after drying, thereby leading to a larger specific surface area.

### 2.5. Particle Size Control of HNS Microspheres

**Figure 7:**
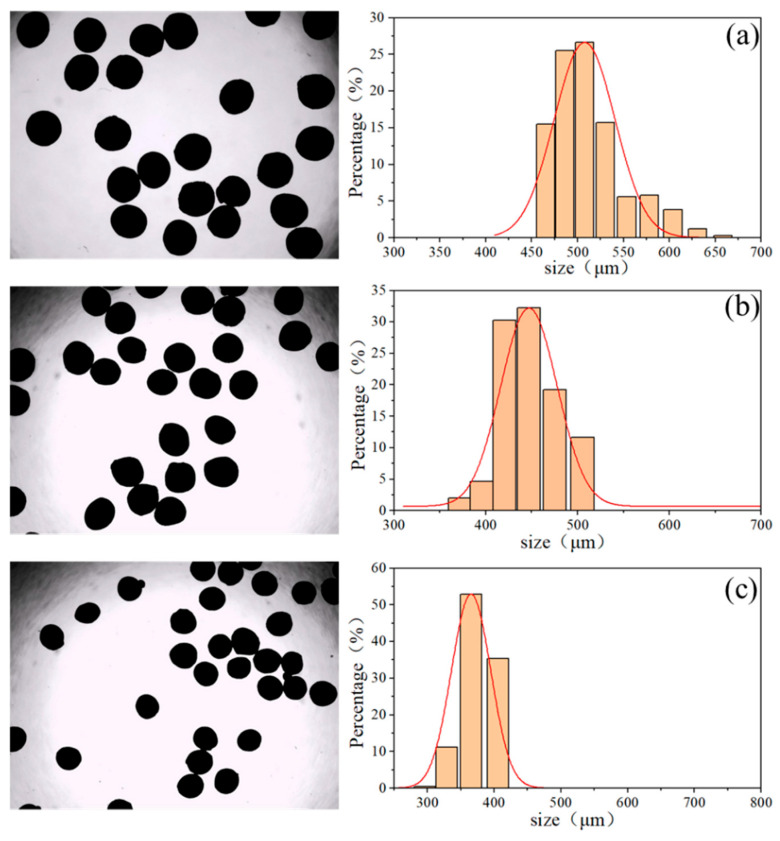
Microscopic photos and particle size determination of microsphere samples (**a**) Q = 1200 μL/min, (**b**) Q = 900 μL/min, (**c**) Q = 600 μL/min. The red lines represent the normal distribution fitting curves for the particle sizes.

As indicated by Equation (5) in Section 2.2, adjusting the driving frequency (*f_p_*) and the fluid flow rate (*Q*) allows for linear control of the droplet volume (*V*). The droplet volume is positively correlated with the final particle size. At a constant driving frequency (*f_p_*), an increase in *Q* inevitably leads to a larger droplet volume, which ultimately leads to an increase in particle size. Based on this principle, under the single-periodic droplet ejection mode, the experiment fixed the driving frequency (*f_p_*) and varied the flow rate (*Q*) to produce HNS microspheres of three different sizes. The particle sizes of the HNS microspheres under various parameters were measured using the BT-1600 image particle analysis system, as shown in Figure 7.

The measured median particle sizes (*D*_50_) of the prepared particles, listed from smallest to largest, were 375.84 μm, 444.45 μm, and 504.22 μm. It can be observed that the particles obtained under different parameters exhibit narrow size distributions and regular spherical structures. However, relatively larger particle sizes are accompanied by broader size distributions. From an energy perspective, a higher energy input tends to destabilize the system. As the flow rate increases, the relative reduction in the airflow’s shear capability on the droplets leads to variations in the final droplet volumes, consequently resulting in a broader particle size distribution. To maintain better size consistency during particle size control, it is essential to simultaneously increase the air pressure (shear intensity) when raising the flow rate. Furthermore, the testing system provided average aspect ratio (*L*/*W*) and circularity information for the three sets of samples, enabling a multi-dimensional understanding of the microspheres’ morphological structure. The structural parameters of the microsphere samples are summarized in Table 1.

To ensure the statistical robustness of the proposed method, it is important to note that the data acquired by the BT-1600 image particle analysis system represents a massive statistical ensemble of thousands of individual microspheres collected during steady-state operation. The “span” parameter shown in Table 1, defined as (*D*_90_ − *D*_10_)/*D*_50_, serves as a direct quantitative measure of the statistical dispersion of the droplet generation process. The obtained span values are exceptionally low (ranging from 0.15 to 0.19), indicating a highly monodisperse size distribution. Furthermore, the average roundness (≈0.89) and length-to-width ratio (*L*/*W* ≈ 1.05) show minimal morphological variance among the particle population. Collectively, these narrow intra-batch statistical distributions rigorously confirm that the single-periodic pulsed air-jet mode essentially eliminates the random volume fluctuations typically seen in passive continuous-flow systems, thereby achieving highly precise and reproducible control over the energetic microsphere granulation.

### 2.6. Thermal Performance Analysis of HNS Microspheres

**Figure 8:**
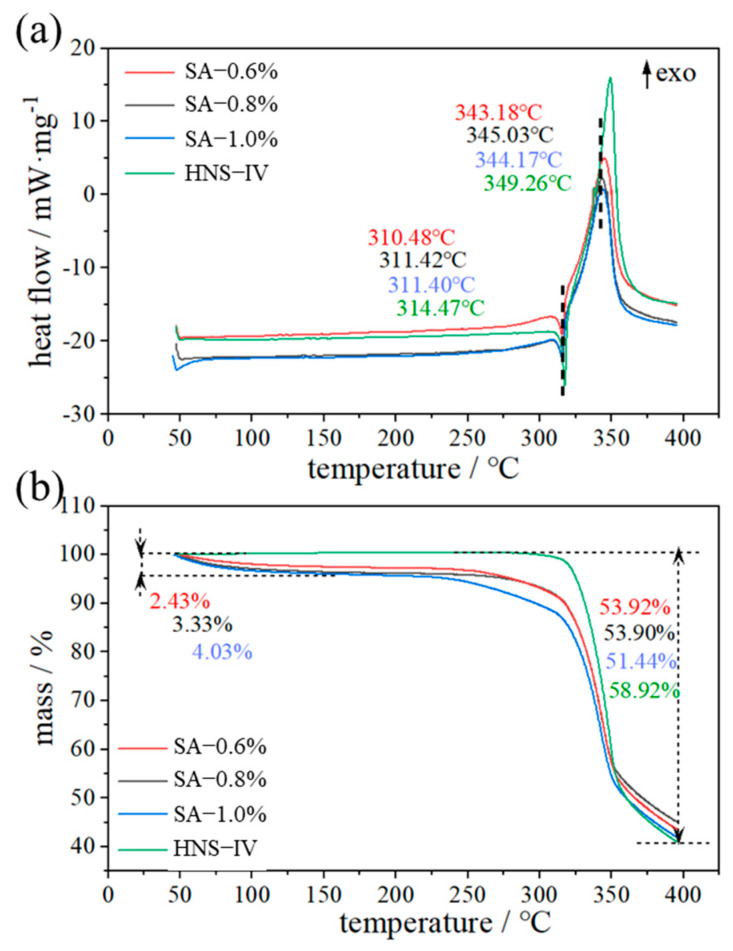
Analysis of thermal decomposition performance of microspheres (**a**) DSC, (**b**) TG. The dashed lines indicate the peak temperatures (**a**) and the onset/end temperatures of mass loss (**b**). The arrow in (**a**) indicates the exothermic direction.

As an inert substance, the introduction of CA inevitably influences the thermal decomposition process of the final energetic material. The CA content in the particles was characterized by the SA concentration. Figure 8 presents the TG-DSC curves of HNS microspheres and HNS-IV at different SA concentrations. The DSC curves of the samples are shown in Figure 8a. It can be observed that when the temperature reaches 310 °C, the tested samples begin to exhibit a small endothermic peak, which corresponds to the melting process of HNS. As the SA content increases, the heat release values determined based on the main reaction peak areas are 2632.59 J/g, 2549.56 J/g, and 2529.12 J/g, respectively, all of which are lower than that of HNS-IV (3550.80 J/g). Compared to HNS-IV, the melting onset temperature and exothermic peak of the microspheres are slightly advanced. We hypothesize that the structural encapsulation and the heat capacity of the capsule components might facilitate local thermal accumulation within the system, thereby slightly shifting the onset of the reaction. However, a comprehensive non-isothermal kinetic analysis (e.g., Kissinger or Ozawa methods) will be necessary in future work to fully decouple the thermal barrier effects from the intrinsic decomposition kinetics of these composite microspheres.

The TG curves of the tested samples are shown in Figure 8b. In contrast to the single-step weight loss process of HNS-IV, the microsphere samples demonstrate a certain degree of mass loss during the initial heating stage. In the temperature range of 50 °C to 150 °C, the weight losses of the microsphere samples in the first stage are 2.43%, 3.33%, and 4.03%, respectively, corresponding to the decomposition of calcium alginate and bound water within the microspheres. However, due to the introduction of inert components, the extent of the main reaction in the microspheres is lower than that of HNS-IV. The mass losses of the microsphere samples with increasing SA content are 53.92%, 53.90%, and 51.44%, respectively, all of which are lower than that of HNS-IV (58.92%).

## 3. Materials and Methods

### 3.1. Reagents and Materials

Reagents: Anhydrous calcium chloride (CaCl2, Sinopharm Chemical Reagent Co., Ltd., Shanghai, China; Analytical Reagent grade); sodium alginate (C5H7O4COONa, Sinopharm Chemical Reagent Co., Ltd., Shanghai, China; Analytical Reagent grade, SA); deionized water (EPED-10TJ, EPED Technology Co., Ltd., Nanjing, China); hexanitrostilbene (HNS, purity ≥ 97.2%, Hubei Oriental Chemical Co., Ltd., Xiangyang, China). The particle size distribution of HNS significantly affects its suspension stability in the SA solution. To achieve stable dispersion of HNS in the solution and ensure the reliable operation of the suspension in the microchannel, HNS-IV was recrystallized using a confocal microchannel based on microfluidic technology, with a *D*_50_ of 214 nm.

Analytical Instruments: Metatest E1-G long working distance metallographic microscopy system (Metatest Corporation, Nanjing, China); Mettler-Toledo TGA-DSC 1-1600 simultaneous thermal analysis system (Mettler-Toledo, Greifensee, Switzerland); SU8220 field emission scanning electron microscope (Hitachi High-Tech Corporation, Tokyo, Japan); FASTCAM Mini UX50 high-speed camera (Photron Limited, Tokyo, Japan); TriStar II Plus 3.03 high-throughput surface area and pore size analyzer (Micromeritics Instrument Corporation, Norcross, GA, USA); BT-1600 image particle analysis system (Bettersize Instruments Ltd., Dandong, China).

### 3.2. Experimental Procedure

**Figure 9:**
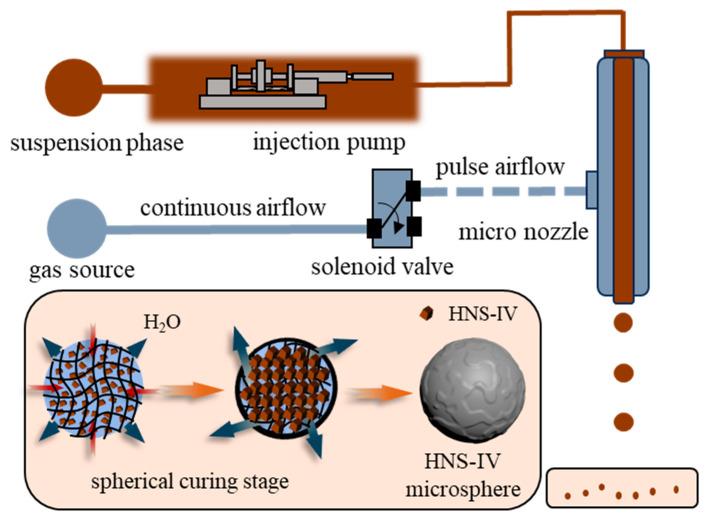
Schematic diagram of droplet granulation system based on pulse airflow shear.

The droplet granulation system based on pulsed airflow shearing consists of a gas source, a pulse control unit, a micro-droplet injection unit, a collection unit, and related connecting components, as illustrated in Figure 9. A signal generator and power amplifier produce pulsed disturbance signals to control the electromagnetic valve, which opens and closes at a specific frequency. The pulsed airflow is generated by shearing the gas flow through the electromagnetic valve. The reagent components are dispersed in an SA solution and injected through the inner tube of a coaxial micro-nozzle, which is regulated by a syringe pump. Simultaneously, the gas flow is introduced into the outer channel at a controlled pressure via a gas pressure valve. Under the shearing action of the pulsed airflow, the reagent suspension is ejected to form micro-droplets. These micro-droplets are collected in a container filled with calcium chloride solution. Through the cross-linking reaction between SA and Ca^2+^ ions, the micro-droplets rapidly solidify into spherical particles. This system enhances the shearing and dispersion effects on the dispersed phase by controlling the flow state under pulsed airflow, enabling the continuous and controllable preparation of HNS-shaped particles.

Specifically, the coaxial micro-nozzle consists of an inner tube (inner diameter: 0.25 mm; outer diameter: 0.5 mm) and an outer gas channel (inner diameter: 0.86 mm; outer diameter: 1.26 mm). The vertical collecting distance from the nozzle tip to the surface of the CaCl_2_ coagulation bath was fixed at 20 cm. The pulsed airflow was precisely modulated by a high-response two-way solenoid valve (response time < 10 ms), and its duty cycle (η) was programmed via a signal generator, which was set to 50% in this work. The air pressure was measured via a pressure gauge in the pipeline upstream of the nozzle. The generated micro-droplets were collected in a 0.8 wt% CaCl_2_ aqueous solution. After collection, the particles were left to stand for 30 min to achieve complete gelation. During the post-treatment process, the microspheres were thoroughly washed with deionized water and ethanol sequentially to completely remove the aqueous solvent and residual Ca^2+^ ions. Finally, to obtain the dried composite microspheres and minimize morphological collapse or shrinkage, the samples were heated and dried under optimized conditions (40 °C for 12 h).

## 4. Conclusions

By constructing a droplet granulation system based on pulsed airflow shear, controlled granulation of HNS-IV was successfully achieved. The effects of SA concentration, pulse frequency (*f_p_*), and flow rate (*Q*) on particle morphology and size were investigated, leading to the following conclusions:

(1) By controlling the pulsed airflow parameters (*P*, *f_p_*, *η*, and *Q*), three droplet ejection modes were realized. Under the conditions of *η* = 50%, *Q* = 600 μL/min, *f_p_* = 30 Hz, and *P* = 2 kPa, droplets were ejected in the single-periodic mode. In this mode, the droplet generation frequency can be controlled by the driving frequency, and the droplet volume can be quantitatively calculated.
(2) Higher SA concentrations result in faster droplet solidification rates, ultimately improving the sphericity quality of the microspheres. The generated microspheres exhibit a porous internal structure, and their specific surface area significantly increases with higher CA content. Based on the N_2_ adsorption–desorption curves, the specific surface areas of HNS microparticles prepared at SA concentrations of 0.6%, 0.8%, and 1.0% were 9.7750 m^2^/g, 11.2250 m^2^/g, and 13.9445 m^2^/g, respectively.
(3) Leveraging the principle of quantitative droplet volume calculation in the single-periodic mode, particle size control was achieved by adjusting Q. Within the studied parameter range, the smallest particle size (*D*_50_) was 375.84 μm, while the largest was 504.22 μm.
(4) Compared to HNS-IV, the introduction of CA influenced the thermal decomposition behavior of the microspheres. Specifically, this was manifested by an earlier reaction onset temperature, reduced heat release, and decreased total mass loss.

To achieve high-throughput droplet granulation, future research could focus on designing novel valve structures to replace the current solenoid valves, enabling multi-channel pulsed airflow for shear processing of the suspension phase. The droplet preparation technology based on airflow shear significantly reduces the accumulation of volatile organic solvents typical of liquid–liquid biphasic systems. Importantly, the residual aqueous CaCl_2_ coagulation bath and washing liquids are non-toxic and can be safely managed through standard laboratory aqueous waste protocols. Therefore, this technology strongly aligns with the principles of green, safe, and intelligent manufacturing of energetic materials. This technology is versatile and can provide valuable insights for the spherical granulation of other energetic materials.

## Figures and Tables

**Table 1:** HNS microsphere structural parameters.

Samples	D50	Maximumparticle Size/μm	Minimumparticle Size/μm	Average Roundness	Span	L/W
a	504.22	650.41	459.58	0.893	0.19	1.05
b	444.45	508.87	361.45	0.892	0.18	1.06
c	375.84	412.80	332.46	0.896	0.15	1.05

## Data Availability

The original contributions presented in this study are included in the article. Further inquiries can be directed to the corresponding author.
